# Purinergic Signaling in the Hallmarks of Cancer

**DOI:** 10.3390/cells9071612

**Published:** 2020-07-03

**Authors:** Anaí del Rocío Campos-Contreras, Mauricio Díaz-Muñoz, Francisco G. Vázquez-Cuevas

**Affiliations:** Department of Cellular and Molecular Neurobiology, Instituto de Neurobiología, Universidad Nacional Autónoma de México, Boulevard Juriquilla #3001, Juriquilla Querétaro 76230, Mexico; anaicampos.c@gmail.com (A.d.R.C.-C.); mdiaz@comunidad.unam.mx (M.D.-M.)

**Keywords:** purinergic signaling, cancer, tumor microenvironment, immune evasion in cancer, purinergic receptors, ATP, adenosine, ectonucleotidase

## Abstract

Cancer is a complex expression of an altered state of cellular differentiation associated with severe clinical repercussions. The effort to characterize this pathological entity to understand its underlying mechanisms and visualize potential therapeutic strategies has been constant. In this context, some cellular (enhanced duplication, immunological evasion), metabolic (aerobic glycolysis, failure in DNA repair mechanisms) and physiological (circadian disruption) parameters have been considered as cancer hallmarks. The list of these hallmarks has been growing in recent years, since it has been demonstrated that various physiological systems misfunction in well-characterized ways upon the onset and establishment of the carcinogenic process. This is the case with the purinergic system, a signaling pathway formed by nucleotides/nucleosides (mainly adenosine triphosphate (ATP), adenosine (ADO) and uridine triphosphate (UTP)) with their corresponding membrane receptors and defined transduction mechanisms. The dynamic equilibrium between ATP and ADO, which is accomplished by the presence and regulation of a set of ectonucleotidases, defines the pro-carcinogenic or anti-cancerous final outline in tumors and cancer cell lines. So far, the purinergic system has been recognized as a potential therapeutic target in cancerous and tumoral ailments.

## 1. Purinergic Signaling in Brief

**Figure 1:**
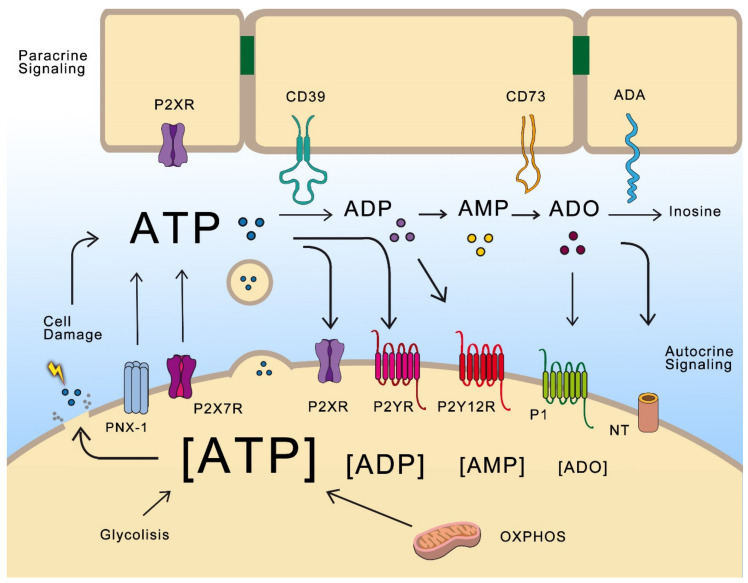
Nucleotides act as autocrine and paracrine messengers. ATP is produced by oxidative phosphorylation (OXPHOS) and glycolysis intracellularly reaching mM concentrations. It can be released to extracellular space by cellular lysis, exocytosis, transporters, hemichannels of pannexin-1 (PNX-1) and P2X7R. Once located at the extracellular space, ATP activates P2XR (ligand activated ion channels), P2YR receptors (belonging to GPCR superfamily), and it can be hydrolyzed by ectonucleotidases (here, CD39 and CD73 are illustrated by their relevance in cancer) to form ADP, AMP and adenosine (ADO). ADP is able to activate P2Y12R and ADO activate G-protein coupled receptor (GPCR) receptors of the P1 family named (A1R, A2AR, A2BR and A3R). ADO is hydrolyzed by adenosine deaminase (ADA) to inosine or it is transported into the cell by nucleoside transporters (NT).

In 1929, Drury and Szent-Györgi provided the first experimental evidence that adenine nucleotides function as signaling molecules. However, the term “purinergic”, and ATP as a signaling molecule, was first proposed in 1972 by G. Burnstock [1]. Although his work was controversial, today it is well recognized that ATP, other nucleotides (adenosine diphosphate (ADP), UTP, uridine diphosphate (UDP)) and ADO are cellular messengers that modulate diverse signaling pathways and participate in physiological and pathological processes, mainly through specific membrane receptors (Figure 1).

Purinergic receptors have been classified into two families: P1, sensitive to ADO; and P2, sensitive to adenine and uridine nucleotides. P1 belongs to the G-protein coupled receptor (GPCR) superfamily, while P2 is divided in two subfamilies. The first is P2X, which are ligand-gated cation channels formed by homotrimeric or heterotrimeric complexes of known subunits (P2X1-P2X7). ATP is the natural ligand for P2X receptors. When activated, these receptors promote rapid depolarization associated with Ca^+2^ and Na^+^ influx, and K^+^ efflux [2]. The second subfamily is P2, and eight P2Y subtypes have been described in mammalian cells: P2Y1, P2Y2, P2Y4, P2Y6 and P2Y11-14. These receptors can be activated by ATP (P2Y2 and P2Y11), ADP (P2Y1, P2Y12 and P2Y13), UTP (P2Y2 and P2Y4), UDP (P2Y6) and UDP-glucose (P2Y14). P2Y2, P2Y4 and P2Y6 are coupled to Gq proteins; thus, their activation leads to phospholipase C (PLC) activation, turnover of phosphoinositides and Ca^+2^ mobilization. P2Y12, P2Y13 and P2Y14 are coupled to Gi proteins producing adenylate cyclase (AC) inhibition [3]. Once in the extracellular space, ATP can either activate P2R or be further dephosphorylated/hydrolyzed by a set of enzymes called ectonucleotidases (Figure 1). There are four families of these enzymes: ectonucleoside triphosphate diphosphohydrolases (NTPDases), ecto-59-nucleotidase (CD73), ectonucleotide pyrophosphatase/phosphodiesterase (ENPP) and alkaline phosphatases (AP) [4].

These enzymes, besides limiting ATP signaling, produce additional ligands for P2Y receptors like ADP to P2Y12, and adenosine to A2-AR (A2-adenosine receptors). Extracellular adenosine (exADO) can activate P1 receptors which belong to a family of GPCRs. According to their sequence and signaling properties, P1 receptors are designated A1R, A2AR, A2BR and A3R. A1R and A3R are mainly coupled to the Gi/o subunit and thus inhibit AC and cAMP production; A2AR and A2BR are mainly coupled to the Gs subunit and stimulate cAMP synthesis through AC activation. Finally, exADO and its associated signaling are regulated by hydrolysis through adenosine deaminase (ADA) and transported into the cell by nucleoside transporters (NTs) [5]. 

When cells are damaged or stressed by changes in osmotic pressure and mechanic deformation, they respond by releasing ATP to the extracellular medium. Aside from this unspecific mechanism, ATP can be released by controlled mechanisms in response to different stimuli. These mechanisms include efflux through membrane channels and transporters (e.g., connexins, pannexins, maxi-anion channels, volume-regulated channels, and ATP-binding cassette (ABC) transporters), purinergic receptors (e.g., P2X7R), and vesicle-mediated release [6]. Purinergic signaling is flexible and adaptable. Released ATP activates paracrine and autocrine communication and, as previously mentioned, its hydrolysis generates a cascade of additional signaling molecules. Almost every cell type expresses a dynamic set of purinergic receptors and ectonucleotidases; therefore, the final outcome depends on a variety of factors, including specific receptors and ectonucleotidases expressed by the cell, as well as the constant fluctuations in the proportion of extracellular and intracellular levels of ATP and ADO.

## 2. Purinergic Signaling and Cancer Hallmarks

### 2.1. Purines in Tumor Microenvironment

Intense efforts have been made to systematize the complex organization of cancer cells within the tumor and the interactions of these cells with the organism [7,8]. An essential concept to understand the principles of this organization is the tumor microenvironment (TME). The TME consists of all interactions between cancer cells and non-malignant cells, such as endothelial, fibroblast and immune cells. The structural, cellular and biochemical composition of this enclosed space modulates cancer cell metabolism, migration and proliferation. It also influences the host immune response [9]. Studying the cellular and molecular composition and interaction of these regions has become increasingly important in the field of pathology, because the downstream effects derived from these interactions could favor tumor growth, invasion and immune evasion.

Purinergic signaling in particular has gained attention in this context, because ATP and ADO are present in high concentrations in the TME [10,11]. Different tumor tissues and cancer cell lines express purinergic receptors and CD39/CD73 ectonucleotidases, generating diverse cellular responses that could depend directly on the cell context and the specific set of purinergic signaling components expressed by the tumor and host cells, known by some authors as the “purinome” [12]. In this section, we will review evidence about the presence of ATP and ADO in the TME.

Extracellular ATP (exATP) and exADO are accepted biochemical markers of cancer, due to their significant levels in the tumor interstitium. ATP release by cancer cells and the subsequent activation of purinergic receptors and intracellular pathways have been reported in various cancer models, such as pheochromocytoma PC-12 cells stimulated with maitotoxin [13]; Ehrlich ascites tumor cells, ATP release induced by mechanical stimulation [14]; A549 human lung cancer cells, by exocytosis triggered by TGF-β stimulation [15]; SKOV-3 ovarian carcinoma-derived cells released by a pipette generated flux [16]; I-10 testicular cancer cells through pannexin-1 [17]. A breakthrough was the monitoring of ATP in vivo in a tumor-bearing mouse with the use of reporter cells carrying an extracellular ATP sensor. ATP was within a low nM range in healthy pericellular space, but increased to high μM levels in the tumor stroma and vicinity [18]. This observation had significant relevance, because exATP is a putative direct source of exADO. Although ADO has not been measured within tumors using an in vivo approach, it was reported that exADO was more abundant in microdialysates from tumoral core regions [19]. However, some conditions in the tumor, such as hypoxia, favor ADO formation. It is well known that, in tumor growth, there is an oxygen gradient. The areas at the center of the cellular mass are hypoxic; in this condition, CD39 and CD73 expression is induced by hypoxia inducible factor 1α/β (HIF-1α/β), and ADO formation is promoted [20,21,22,23].

Furthermore, the ectonucleotidases CD39 and CD73 play a fundamental role in modulating ATP and ADO levels in the TME. These enzymes are expressed in cancer cell lines, immune cells and stromal cells, and they are considered immune checkpoints in cancer [24]. Practically all cell types can release ATP to the extracellular space; therefore, all cells in the tumor-host interface could contribute to the substantial amount of ATP in the tumor interstitium. 

A mechanism that has gained attention in cancer is ATP release through pannexin-1 channel (PANX1), since a truncated PANX1 protein (PANX1^1−89^) is significantly enriched in highly metastatic human cancer cell lines [25]. PANX1^1−89^ in combination with wild type PANX-1 confers gain-of-function to channel activity, promoting a significant increase in ATP release. Moreover, this mechanism facilitated the resistance to mechanical deformation of cancer cells. This finding is relevant, because many cancer cells undergo apoptosis during metastasis through capillaries [25]. As previously mentioned, ATP can also be released through P2X7R channel, and the P2X7R has been associated with the regulation of NLRP3 inflammasome, which leads to the release of pro-inflammatory cytokines, specifically IL-1β and IL-18 [26,27,28]. Importantly, antitumor therapies like chemotherapy or radiotherapy induce tissue damage and cell death and, consequently, the corresponding release of damage-associated molecular patterns (DAMPs), mainly ATP [29]. exATP is quickly converted into ADO by the CD39/CD73 pathway, establishing a particular proportion of purines in the TME where both purines can potentially affect cancer and host cells; this equilibrium is decisive for the outcome of a given clinical treatment [30].

Nucleotides in the TME primarily serve as an interface of interaction with immune system cells since ATP can act as a “find me” signal for cells of the innate immune system [30]. However, in the extracellular space, ATP is modified by ectonucleotidases that generate ADO, whose fundamental antagonistic action is related to the evasion of an immune attack; this topic will be discussed later. On the other hand, nucleotides in the tumor stroma can function as paracrine-autocrine messengers, inducing specific cellular responses over all the cell types forming the tumor mass. Research has demonstrated that purines can regulate cell proliferation, epithelial to mesenchymal transition (EMT) and cellular migration in tumor cells. These actions are discussed below. 

### 2.2. Purines in Proliferation and Tumor Growth

Nucleotides in the tumor microenvironment play a dual role; they function as lures to interact with immune system cells and as autocrine-paracrine signals directly affecting the physiology of cancerous cells.

**Figure 2:**
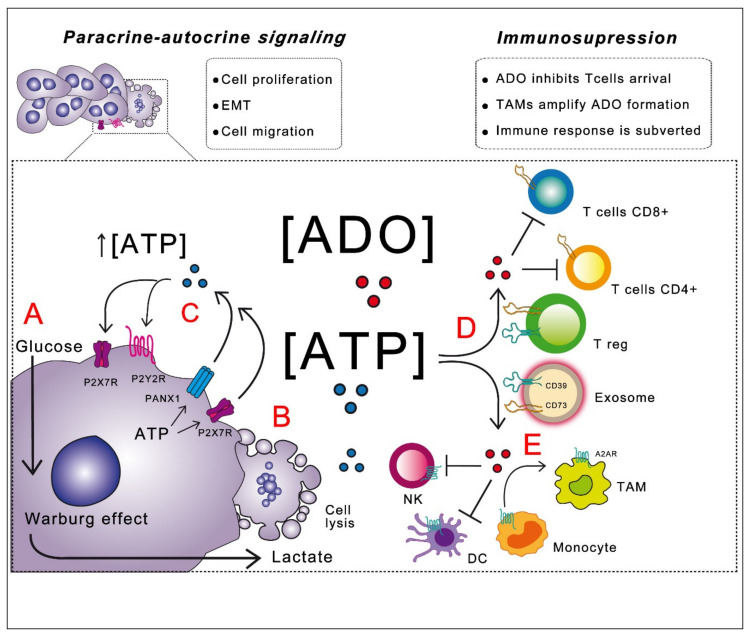
Purinergic signaling and tumor microenvironment (TME). (**A**) Cancer cells synthetize ATP rather from aerobic glycolysis (Warburg effect) which leads to lactate formation and subsequent extracellular acidification. (**B**) ATP is released from tumor cells (pannexin-1 hemichannels and P2X7R have an outstanding role) by general mechanisms or by cellular lysis as result of anticancer therapies and reach hundreds of mM levels, sufficient to activate any P2 receptor. (**C**) extracellular ATP in autocrine/paracrine way activate P2 receptors (mainly P2X7R and P2Y2R) to induce proliferation, migration and epithelial to mesenchymal transition (EMT) of cancer cells. (**D**) In TME, ATP is hydrolyzed to ADO by subsequent action of ectonucleotidases CD39 and CD73, which are expressed in the own tumor cells, exosomes and immune cells (i.e., CD4+, CD25+, Foxp3+ Treg); contributing to the significant increase of ADO, which in turn inhibits antitumor response of innate immune cells and T effector cells (CD4+ and CD8+). (**E**) ADO also contributes to monocyte differentiation into associated tumor macrophages 2s), which also amplify ADO formation.

Autocrine-paracrine actions of purines involve a feedback loop that joins the initial production and release of ATP by cancerous cells, with the subsequent activation of cell proliferation and tumor growth (Figure 2). Signaling actions of exATP depend on the presence and diversity of specific receptors in the own cell releasing the nucleotide and neighboring cells, as well as on the actions of ectonucleotidases, which will define the composition of ligands in the medium.

With respect to purinergic receptors, P2X7R is the best characterized in the cancer context, probably because this receptor was described as an apoptotic inducer [31], motivating the inquiry of a role in cancer. In addition, P2X7R has diverse signal transduction mechanisms. Unlike other P2XRs, it has a long intracellular COOH-end with putative protein-protein interaction domains, such as SH2, SH3 and dead domains [32], creating a potential signaling mechanism independently of ionic conductance. 

Incremented expression of P2X7R has been demonstrated in cancerous tissue from organs including breast [33], thyroid [34], ovary [16], pancreas [35], colon [36,37] and liver [38]; in general, the increment in P2X7R expression was correlated with a high tumor grade. This observation suggests that P2X7R can be activated by autocrine-paracrine signaling and be a regulator of cancerous cell physiology.

The significance of the elevated expression of P2X7R was intriguing, because it was initially related to apoptosis induction; however, important observations supporting proliferation and/or survival roles for P2X7 were later reported; DiVirgilio’s group proposed that P2X7R can act as a growth-promoting receptor based on the following evidence: 1) P2X7R exogenous expression in several cell lines incremented proliferation; 2) the TME contains high amounts of ATP (hundreds of μM) to activate P2X7R; 3) various cancerous tissues from different organs show high expression levels of P2X7R; and 4) P2X7R is a positive regulator of aerobic glycolysis [39]. This apparent antagonism between the ability of P2X7R to induce apoptosis and, in some conditions, support cell survival has been analyzed [40]. The most plausible explanation to explain this paradox could be related with the conformation of P2X7R in activated state, P2X7R adopt structure conformations that specifically regulate the induction of apoptotic activity [41].

The proliferative role of P2X7R has been documented in a variety of cancers, such as ovarian carcinoma cells [16], mesothelioma [42], pancreatic cancer cells [35,43] and osteosarcoma cells [44]. Although the molecular mechanisms of P2X7R are not completely understood, their transduction pathways involve ERK phosphorylation of both dependent and independent intracellular Ca^2+^ increments [45,46], the PI3K/AKT/GSK3β/β-catenin pathway and mTOR/HIF1α/VEGF signaling [16,43]. 

The overexpression of purinergic P2Y receptors belonging to the GPCR superfamily has also been observed in cancerous systems acting as promotors of cell proliferation; P2Y2R is the prototypical and most analyzed receptor. It has been shown that P2Y2R is overexpressed in biopsies of basal cell and squamous cell carcinomas (non-melanoma skin cancers) [47]. One study also showed by cDNA microarray analysis that the P2RY2 transcript is highly expressed in fresh biopsies of gastric cancer tissue, compared to adjacent healthy tissue [48]. The incremented expression of P2Y2R was detected in primary cultured hepatocellular carcinoma cells and in the hepatocarcinoma-derived cell lines HepG2 and Bell-7404 compared with normal hepatocytes and the normal hepatocyte cell line LO2 [49].

Accordingly, it has been demonstrated that UTP activation of P2Y2R induced proliferation in C6 glioma cells [50], in human cutaneous squamous cell carcinoma lines (A431) [47]; in pancreatic duct epithelial cells PANC-1 [51]; in hepatocarcinoma cell lines HepG2 and Bell-7404 [49]; and in the gastric cancer lines AGS and MKN-74 [52]. In addition, P2Y2R-dependent cell proliferation involves the Ras/Raf/MEK-1 pathway, modulated by PLC/PKC and Ca^2+^ in C6 glioma cells [50]. In PANC-1 cells, UTP increased the phosphorylation level of AKT through PKC, PI3K, SRC and Ca^2+^-calmodulin-dependent protein kinase II [51].

In addition, gastric cancer cell lines also express P2X4R, the activity of which exerts anti-proliferative effects contrary to P2Y2R activity [52]. In fact, P2X4R activity is able to revert the proliferative effects mediated by P2X7R in breast-derived cancers [53]. Both evidences reveal that functional interactions among subtypes of purinergic receptors are determinants for the final outcome of purinergic signaling in cancer; both observations highlight the anti-proliferative action of P2X4R.

Although the purinergic system has mainly been associated with the positive regulation of cell proliferation, diverse evidence supports that it is not a rule; for example, pharmacological activation of purinergic receptors induced apoptosis in cancerous cells. Thus, it was shown that P2X7R is downregulated in endometrial cancer. In endometrial epithelial carcinoma cells, P2X7R activation was able to induce apoptotic cell death [54]. Furthermore, activation of P2X7R inhibited the formation of virus-induced skin cancer in vivo [55]. As for P2YR, research has found that P2Y2R activity inhibits cell proliferation in endometrial carcinoma cells HEC-1A and Ishikawa cells [56], human colorectal carcinoma cell HT29 and Colo320 D [57], human esophageal cancer cells [58], and nasopharyngeal carcinoma cells [59]. Inhibition of cell proliferation was also related to the activity of P2Y6R through a pathway involving the store-operated Ca^2+^ entry (SOCE) and β-catenin [60]. These controversial observations must be analyzed considering the detailed characteristics of each cellular system.

The most relevant compound formed from exATP is ADO. The receptors for ADO are expressed in tumor tissues from various organs. It has been shown that A2BR, probably the best characterized in cancer, is overexpressed in tumor biopsies and cell lines derived from human hepatocellular carcinoma [61], colorectal carcinoma [62], oral squamous carcinoma [63] and bladder urothelial carcinoma [64]. To further support the role of A2BR in cancer progression, studies have shown that pharmacological or genetic inhibition of this receptor decreases cell proliferation [62,63,64]. Additionally, expression of A2BR has been described in cell lines derived from prostate cancer [65], breast cancer [66] and head and neck squamous cell carcinoma [67]. In all these systems, A2BR functioned as a cell proliferation promoter. Moreover, inhibition of A2BR expression in EJ and T24 cell lines, derived from bladder urothelial carcinoma, inhibited cell proliferation and arrested the cells in the G1 phase of the cell cycle [64].

Given that the equilibrium between nucleotides and nucleosides influences the autocrine-paracrine signals that regulate tumor growth, purinergic signaling elements, such as transporters, receptors and ectonucleotidases, emerge as potential pharmacological targets to modulate carcinogenesis.

### 2.3. Purines in Cancer Cell Migration, EMT and Metastasis

Cell migration involves a response to a chemical gradient and is required for physiological events including embryonic development and tissue repair; however, in cancer, it participates in the metastatic activity of cancerous cells to form secondary tumors. The stages of metastasis are loss of cell-cell adhesion in primary tumor, migration and invasion, anoikis evasion and implantation to form the secondary tumor [68]. These stages are initiated by epithelial-mesenchymal transition (EMT), a process in which epithelial cells assume a mesenchymal phenotype, acquiring enhanced invasive and metastatic capacity. Extensive evidence indicates that purinergic signaling participates in the modulation of this phenomenon in different cancer types [69].

During EMT, cells lose their apical-basal polarity and epithelial cell-cell contacts including tight junctions, adherent junctions, and desmosomes. In addition, they acquire a spindle-shaped mesenchymal morphology, and gain motility by reorganizing their actin cytoskeleton. EMT is also accompanied by the loss of epithelial genes such as E-cadherin, keratins and zona occludens-1 (ZO-1). Conversely, the expression of metalloproteinases (MMPs), vimentin and N-cadherin is upregulated. Some classical EMT promoters are transforming growth factor β (TFG-β), epidermal growth factor (EGF) and wingless (WNT); these molecules elicit EMT through the activation of transcription factors such as SNAIL and TWIST [70]. ATP and purinergic signaling also modulate the EMT process, migration/invasion and metastasis in many different cancers.

It has been observed in different lung cancer cell lines that stimulation with high ATP concentrations, such as those found in the TME (0.5–1 mM), favors cell detachment, migration and invasion. These observations were associated with an increased expression of MMPs and the formation of filopodia and cell protrusions, as well as an increased expression of vimentin, SNAIL and SLUG. In parallel, there was a reduction of the epithelial proteins E-cadherin and ZO-1. These results were ingeniously related with the exATP micropinocytosis process, since the genetic deletion of SNX5 (a gene involved with cell micropinocytosis) caused a significant reduction in cancer cell proliferation, migration and invasion [71].

Moreover, evidence reveals the interaction between ATP and classical EMT inducers. For instance, it has been demonstrated that treatment with TGF-β1 elicits ATP release from lung cancer cells, thus activating P2 receptors. Actin remodeling and cell migration induced by TGF-β1 required the expression and autocrine stimulation of P2X7R, since these processes were suppressed after P2X7R knock-down or pharmacological inhibition [15]. In the PC9 human lung cancer cell line, which has a mutated EGFR, P2X7R was constitutively activated, promoting cell migration, even in the absence of TGF-β1. Cell motility and lamellipodium extension of PC9 cells were abolished by AG1478, an EGFR inhibitor. These data showed a cross-signaling between TGF-β1, P2X7R and EGFR in the regulation of cell migration [72]. 

P2X7R is associated with cancer cell migration and invasion. This receptor is expressed in cells from different types of cancer, such as pulmonary [15,70], prostatic [73], mammary [74], pancreatic [35], glioma [75], osteosarcoma [44] and glioblastoma stem cell cancer [76]. In the prostate, breast and osteosarcoma cell lines, it has been proven that P2X7R stimulation induces cell migration and up-regulation of EMT-related genes. At the same time, E-cadherin is down-regulated. These effects of P2X7R were mediated through PI3K/AKT phosphorylation and ERK1/2 signal transduction pathways [71,74].

Considering that nucleotides promote cell migration, evidence demonstrates the interaction between purinergic receptors and proteins involved in cell-to-cell and cell-to-extracellular matrix (ECM) junctions such as cell adhesion molecules (CAM) and integrins. For instance, P2Y2R interacts directly with αvβ3 and αvβ5 integrins in astrocytoma cells. These interactions are mediated through the integrin-binding domain arginine-glycine-aspartic acid (RGD) contained in P2Y2R. The RGD domain is necessary for UTP-induced chemotaxis through G_0_ protein coupling; the mechanism elicited by P2Y2R stimulation involves Rac and Vav2 (a GEF for Rac) activation. Moreover, vitronectin, an ECM protein that binds to integrins αvβ3 and αvβ5, is up-regulated [77]. Additionally, P2Y2R activation through G_12_ coupling and integrin αvβ5 interaction mediates Rho activation, cofilin, myosin light chain (MLC-2) phosphorylation and stress fiber formation [78]. One study showed that P2Y2R activation increased intracellular cell adhesion molecule-1 (ICAM-1) and vascular cell adhesion molecule-1 (VCAM-1) expression in a highly metastatic breast cancer cell line. This effect was also observed in endothelial cells incubated with cancer cell conditioned medium, leading to increased adhesion between cancer cells and ECs; this action could be associated with cancer cell metastasis [79]. 

P2Y2R is a purinergic receptor that seems crucial to mediate ATP pro-metastatic effects. For instance, ATP in prostate cancer cells promotes Cdc42 and Rac1 activation and MMP expression through P2YR activation [80,81]. This effect is mediated through P2Y2R activation [82]. P2Y2R is also expressed in diverse breast cancer cell lines: MCF-7, Hs578T, MDAMB-231 and T43D [83,84,85]. In breast tumor tissue, P2Y2R expression is higher at the invasive edge of the tumor, in infiltrating cells in adipose mammary tissue and in the tumor embolus in lymphatic sinuses, suggesting the participation of P2Y2R in metastasis [84]. It has been proven that highly metastatic breast cancer cell lines release more ATP to the extracellular medium and, thus, exhibit a greater ability to migrate and invade [86], the effects are mediated through the activation MEK/ERK1/2-dependent signaling pathway [80,83,87]. Another pathway involved in cell invasion of breast cancer cells is ATP-P2Y2R-β-catenin [85]. Studies in prostate [87] and ovarian cancer cells [88] found that P2Y2R activation also promoted the expression of EMT-related genes, and demonstrated a transactivation pathway between P2Y2R and EGFR. 

Conversely, CD73 over-expression using pcDNA-NT5E has shown to increase cancer cell invasion, migration and adhesion in the breast cancer cell lines T-47D and MDAMB231 [89,90]. Following the same experimental strategy, increased cell migration was observed in human cervical cancer cell lines. However, the effect did not depend on CD73 activity [91]. In contrast to the general assumption that CD73 is pro-tumorigenic, it was reported that CD73 promotes epithelial integrity through an increase in membrane E-cadherin, β-catenin and Na^+^-K^+^ ATPase in endometrial cancer; also, in vitro experiments showed increased migration and invasion after pharmacological CD73 inhibition [92]. 

Analysis of CD73 expression in human tissue from head and neck squamous cell carcinoma (HNSCC) samples showed a higher CD73 expression in samples from patients with lymph node metastasis. This finding correlated with in vitro experiments, in which, after CD73 knock-down, cancer cell migration and expression of EMT-genes were reduced and A3R activation promoted HNSCC cell migration and presumably involving the EGFR signaling pathway [93]. In ovarian cancer cells, CD73 confers stemness and the expression of EMT-associated genes [94]. 

CD73 expression in hepatocellular carcinoma is correlated with a mesenchymal phenotype. CD73 activity was required for inducing mesenchymal characteristics. A2AR activation could restore the effect of knocking down CD73. These data suggest a synergist treatment with A2AR and CD73 inhibitors [95].

Activity of CD73 produces ADO and the potential activation of P1 receptors. Virtanen et al. in 2014 [96] demonstrated that ADO at low µM inhibited cell migration and invasion in prostate and breast cancer cell lines. However, the authors suggested that these effects were not mediated by the activation of P1 receptors, but by one intrinsic receptor-independent mechanism. However, the inhibitory effect of ADO in cell migration and invasion has also been proven in human cervical and ovarian cancer cell lines [89,97].

Despite the opposite effects regarding ADO modulation in cancer cell migration and invasion, it is necessary to consider the receptor involved and the type of cancer under study. For instance, pharmacological A1R inhibition reduces cell migration in renal cancer cell lines [98]. On the contrary, gastric cancer cell incubation with ADO enhances the expression of stemness and EMT genes, which is attributed to A2AR activation and the AKT-mTor pathway [99]. The effect of A2BR on EMT has been evaluated in human epithelial lung cancer cells; interestingly, two modulatory roles were described. The first consisted in a partial EMT induction through A2BR activation that involved the cAMP/PKA and MAPK/ERK transduction pathways. The second consisted in the ability of the selective A2BR agonist, BAY-606583, to counteract TFG-β–induced EMT [100]. These roles suggested that EMT maintenance/inhibition is based on the balance of extracellular environment signals. In agreement, in MDAMB231 cancer cells, ADO increased cells migration through the A2BR/AC/PKA/cAMP axis [66] and A2BR pharmacological inhibition decreased cell migration in human epithelial lung cancer cells and renal cancer cell lines [67,101]. Finally, A3R modulation in cell migration has been evaluated in AT6.1 rat prostate [102], MDA-MB-23 human breast [103], HepG2 hepatocellular and Caco3 colorectal cancer cell lines [104]. These reports demonstrated that A3R stimulation arrested cancer cell motility, migration and invasiveness by hindering AC/PKA and reducing NADPH oxidase activity [102]. On the other hand, in primary cultures of glioblastoma (GBM) stem-like cells obtained from GBM patients, and also in a GBM cell line, A3R blockade promoted a reduction in cell migration and invasion associated with the expression of EMT genes [105].

Evaluating ATP cellular regulation and consumption during metastatic cell migration is essential, considering that ATP is the cell’s biological energy currency. This has been elegantly achieved by Zanotelli and colleagues, who used genetically encoded fluorescent biomarkers to evaluate cancer cell migration in 3D matrices. They found that the ATP:ADP ratio was modulated in response to collagen architecture. This ratio increased in denser matrices where migration is impaired and decreased in aligned matrices where migration is facilitated. Therefore, the cellular energy requirement changes in response to the adhesion environment. It can be suggested, however, that increases of ATP in the TME could facilitate cancer metastasis [106]. Additionally, striking evidence has shown that ATP is required locally in invadopodia formation. It participates in F-actin network growth, even in the absence of MMPs [107]. These data indicate that ATP per se engages in the physiology of cell migration and metastasis.

### 2.4. Energy Metabolism in Cancerous Cells

Cancer is a pathology with multifaceted expression. Neoplastic conditions that cause tumor growth involve a variety of molecular, cellular and metabolic adequacies. Foremost among them are biochemical reactions, considered hallmarks of cancer, especially in the form in which cancerous cells display energy transformations in cytoplasmic and mitochondrial compartments. Almost 100 years ago, Nobel Laurate Otto H. Warburg described that carcinogenic cells obtain ATP preferably from glycolysis, regardless of the availability of oxygen and the suitability of mitochondrial oxidative phosphorylation [108]. This “aerobic glycolysis” or “Warburg effect,” as it was rapidly known, was a widely spread metabolic feature in many tumor-derived cells and cancerous cell lines [109,110].

However, there is an accepted rationale for the Warburg effect in the biology of cancer. It has been more difficult to reach consensus regarding the metabolic mechanisms that sustain this neoplastic energy adaptation. Indeed, cancerous cells are systems specialized in cellular growth and duplication. The more undifferentiated and aggressive the cancer cells, the more prone they are to activate their cellular cycle and the metabolic pathways to synthesize biomolecules and build new genetic material and phospholipidic membranes [111]. Therefore, growing tumors and carcinogenic cells face a “metabolic dilemma”; that is, deciding what is more important in a replicating system: 1) cellular energy like ATP to enable the biosynthetic processes, or 2) the availability of biomolecules, such as reductive power (NADPH), fatty acids, amino acids, glycerol and sugars, to be used as structural elements for the synthesis of membrane and genetic material. In this context, understanding the implications of the complex metabolic adaptations associated with cancer is necessary to visualize successful therapeutic approaches [112].

Metabolic reprogramming in cancerous cells does is not just an imbalance between cytoplasmic glycolysis and mitochondrial oxidative metabolism. Genetic activation of glycolytic-promoting factors, such as c-Myc and HIF-1α (transcriptional factors), glucose transporters and glycolytic enzymes and regulators (hexokinase 2, pyruvate kinase M2, pyruvate dehydrogenase kinase isozyme 1 and lactate dehydrogenase A) underlie an enhanced glycolytic flux associated with aerobic glycolysis [113]. The immediate consequences of this enhanced glycolytic flux include increased glucose uptake with concomitant glycogen formation, as well as extra-cellular acidification connected to prominent lactate production.

Another metabolic flux that is activated in cancerous cells is the pentose phosphate pathway (PPP). When glucose is metabolized by the PPP, it promotes the synthesis of 5-carbon sugars used in the polymerization of nucleic acids, but most importantly, it favors the formation of the redox coenzyme NADPH. This cofactor is key for various anabolic pathways such as lipogenesis (β-reduction) and isoprenoid/sterol synthesis; in addition, NADPH is necessary to maintain functional levels of the antioxidant glutathione in its reduced form (GSH) [114].

The increased glutamine metabolism that is characteristic of neoplastic cells is also part of the adaptations associated with the Warburg effect. In this case, glutaminase catalyzes the conversion of glutamine into glutamate. Glutamate, by action of the glutamate dehydrogenase located within the mitochondria, loses ammonium molecules and forms α-ketoglutarate. α-Ketoglutarate is an intermediate of the Krebs cycle, which acts as a redox substrate, to form NADH and supply oxaloacetate. Overall, glutamine is used by cancerous cells as an anaplerotic substrate by the coordinated action of cytoplasmic and mitochondrial enzymes [115].

Mitochondrial citrate is also crucial in cancer metabolism. Citrate is constantly leaving mitochondria to enter the cytoplasm and be converted into the lipogenic substrate acetyl-CoA by the activity of ATP-citrate lyase. Acetyl-CoA acts as a substrate for the formation of fatty acids, which are incorporated into phospholipids and triacylglycerols. Citrate exits the mitochondria, so the mitochondrial role of glutamine metabolism is relevant for aerobic glycolysis: the amino acid contributes as a carbon skeleton to supply the carbons lost by the exit of the mitochondrial citrate [116]. It has been reported that mitochondrial activities during carcinogenesis, such as ATP production, glutamine metabolism, fusion/fission balance and calcium dynamics, are regulated by the metabolic master regulator mTORC1 [117].

Some cellular populations display a Warburg-like effect in the metabolic adaptation, without being cancerous. For example, the functional unit glia-neuron in the nervous system. It has been reported that astrocytes are primarily glycolytic and effective lactate producers. Eventually, the lactate formed by the glia is taken up by the neuron, where it is oxidized as energy substrate. The glycolytic activity in astrocytes occurs whether they possess functional mitochondria and adequate oxygen availability or not [118,119]. 

#### Warburg Effect and Purinergic Signaling

Signal transduction by purinergic receptors has been little explored in the characterization of the Warburg effect and other metabolic adaptations in cancerous cells. Until March 24, 2020, from the total of entries in PubMed focused on the Warburg effect (2693), only 0.7% were related to purinergic signaling (20).

Among the purinergic signaling elements, P2X7R has been the most studied, in relation to the metabolic adaptations that occur in cancerous cells. More than 20 years ago, it was recognized that P2X7R promoted proliferative actions in lymphoid cells [120], in contrast to the pro-apoptotic and necrotic role previously designated to this cationic channel receptor [121]. Growth-promoting effects associated with elevated levels of extracellular ATP and P2X7R activation also involved MAPK/ERK kinases, by inducing de novo synthesis of pyrimidine nucleotides [122]. The pro-mitotic role of P2X7R was also recognized in B-cell chronic lymphocytic leukemia, one of the most common neoplastic diseases in the Western world. P2X7R expression was higher in patients suffering from an aggressive form of this cancer [123]. Tumor progression has also been related to the expression of P2X7R in prostate and breast cancer [33].

A seminal article demonstrated a direct role of P2X7R in the metabolic adaptations that underline the Warburg effect [124]. This group showed that in P2X7R-transfected HEK293 cells and the neuroblastoma cell line ACN, there was an increased lactate output associated with cell proliferation in the absence of serum, a hallmark of aerobic glycolysis. P2X7R action was accompanied by the upregulation of the following glycolytic promoters: glucose transporter Glut1, glyceraldehyde 3-phosphate dehydrogenase (G3PDH), phosphofructokinase (PFK), pyruvate kinase M2 (PKM2) and pyruvate dehydrogenase kinase 1 (PDHK1). Furthermore, P2X7R expression inhibited pyruvate dehydrogenase (PDH) activity, increased phosphorylated Akt/PKB and hypoxia-inducible factor 1a (HIF-1α) expression, and enhanced intracellular glycogen stores. These are all metabolic adjustments to avoid aerobic adaptations.

To accomplish the promotion of the Warburg effect and the proliferative effect independent of serum, P2X7R must reach higher levels of activation to function not just as an ion channel, but as a large conductance non-selective pore. Acting in this way, P2X7R is capable of mitochondrial stimulation by increasing the resting mitochondrial potential (ΔΨ) and the basal mitochondrial calcium [125]. 

The authors of [126] reported that human non-small cell lung cancer A549 showed the capacity to internalize the highly concentrated extracellular ATP by clathrin- and caveolae-mediated endocytosis, but mainly by macropinocytosis. The internalized ATP favored elevation of intracellular energy charge and promoted cancer growth, survival, and drug resistance, as well as the induction of EMT. More recently, these observations were extended to other neoplastic cell lines [71]. In the context of the Warburg effect, an interesting interpretation is that the metabolic role played by the internalized ATP serves as an energy supplement for the glycolytic ATP in cancerous cells. This phenomenon resulted only partially dependent on P2X7R [71].

It was demonstrated in prostate cancer cell lines that activation of the pro-inflammatory Toll-like receptor 3 stimulated the Warburg effect (glucose utilization and lactate production). This effect involved the intracellular participation of HIF-1α, and was synergized by the extracellular activation of A2BR [127].

The high extracellular ATP concentration characteristic of neoplastic cells is also related to the elevated presence of exADO, according to the expression and activity of various ectonucleotidases. In this context, the nucleoside ADO has been recognized as a pro-tumoral factor [128]. For example, in non-small cell cancer tissues and cancer-associated fibroblast, antagonists for A2AR (ZM241385 and SCH5826) inhibited cellular proliferation and the human tumor xenograft in mice [129].

P2XR is also involved in the regulation of metabolic responses and the Warburg effect. P2X1R and P2X7Rs were studied in leukemia T cells (Jurkat) showing that basal activation of both receptors increases the levels of intracellular calcium. Upon pharmacological inhibition of these receptors, Jurkat, THP-1, U-937 and HL-60 cells decrease mitochondrial activity, calcium signaling and cell proliferation. The authors concluded that the coordination of cytoplasmic and mitochondrial energy responses promotes autocrine purinergic signaling and the uncontrolled proliferation of leukemia cells [130].

### 2.5. Purines and Evasion of Immune Attack

Signaling through extracellular nucleotides by tumor cells is relevant for interactions with a host immune system. While exATP elicits a “find me” signal that promotes an innate and adaptive immune response by attracting immune cells. In the tumor context, this response is subverted, mainly by the sequential processing of exATP into ADO by action of the CD39 and CD73 ectonucleotidase pathway; ADO acts as an immunosuppressive molecule directing the phenotype of infiltrated immune cells in the TME dismantling the antitumor immune attack [24,30,131]. Thus, the purinergic molecular code defines the significance and outcome of the interaction between the tumor and the host immune system cells.

In the tissue damage context, cells release DAMPs in response to conditions as hypoxia, inflammation and necrosis. ATP is recognized as a DAMP, since exATP recruits neutrophils, macrophages and dendritic cells (DCs) to contribute to damage resolution [132,133,134]. In cancer, it has been described that the ATP released by dying cells because of anticancer therapies circulates through the TME to activate receptors in the membrane of tumor-infiltrated cells. When P2X7R is activated in DCs, IL-1β is secreted through the P2X7R-dependent assembly of the NLRP-3 inflammasome. IL-1β, a proinflammatory cytokine, induces the immunogenic response associated with CD8^+^ T cells. Thus, anticancer therapy with oxaliplatin and anthracyclines, in p2rx7^−/−^, casp1^−/−^ or nlrp3^−/−^ genetic background was inefficient; these evidences link NLRP-3 inflammasome activity with anticancer therapy treatment efficacy [135,136]. 

Moreover, the relevance of P2X7R expression in tumor-host interactions, specifically immune cell diversity of the TME, has been analyzed, by comparing the identity of immune cells and cytokine expression in the TME of tumors induced by xenotransplantation of murine B16 melanoma cells (a cell line expressing high levels of P2X7R), in both null mice for P2X7R (p2rx7^–/–^) and wild-type animals treated with a P2X7R antagonist (wt_AT_). Tumor growth was accelerated in the p2rx7^−/−^ background. The cells infiltrated in tumor-bearing p2rx7^−/−^ contained an immunosuppressive microenvironment, compared to those growing in the wt_AT_ background, with fewer effector T cells (T_eff_) (CD8^+^ and CD4^+^), increased T_reg_ cells (CD4^+^, CD25^+^, Foxp3^+^) and a decline in cytotoxic effector CD8^+^ T cells (T_cyt_). Additionally, the TME was enriched in pro-inflammatory cytokines, such as IL-1β, IL-18 and IFN-γ. Regarding ectonucleotidases, CD73 was highly expressed, not only in immunosuppressive T_reg_, but also in CD8^+^ T_eff_ and macrophages; CD39 was also elevated in T_eff_. These changes in CD39/CD73 expression produced a reduction in exATP levels in the TME and an increment in ADO production, causing a general immunosuppressive effect. Conversely, the pharmacological blockade of P2X7R in wt_AT_ mice, besides reducing tumor growth, promoted an anti-tumor immune infiltrated with incremented IFN-γ and reduced IL-1β, but without affecting the TME exATP [137]. These results showed that P2X7R expression in the host tissue contributed with an anti-tumor immune response, and confirmed that the deficiency of host P2X7R induces immune failure, suggesting that P2X7R plays a relevant role in the establishment of the immune response in the TME, thus integrating tumor-host interaction.

In contrast, it has been suggested that exATP contributes to immunosuppression in the TME. A study in acute myeloid leukemia showed that exATP, induced by chemotherapeutic agents, promoted the up-regulation of T_reg_ cells [138].

Taken together, this evidence indicates that ATP mediates the interaction of tumor cells with components of the immune system and modulates the inflammatory state in the TME. On the other hand, a common mechanism of purine actions in TME is the formation of ADO, which will be discussed next.

It has been established that hypoxia, aside from generating a protective environment for tumor cells, is the main cellular condition favoring ADO accumulation in the TME. Hypoxia increments the expression level of the ectonucleotidases CD39 and CD73 in a way that depends on HIF-1 transcription factor activity [20,21,22,23]. ADO inhibits T cell arrival in the tumor through the activation of its receptors, thus preventing these cells from producing their cytotoxic activity against cancer cells. It has been proven that the main P1 involved in immunosuppression is A2AR, since its genetic deletion facilitates tumor rejection by T cells [19]. These observations are supported by findings in which supplemental oxygenation (hyperoxia) facilitated tumor regression, enhanced tumor infiltration of CD8^+^ T cells, reduced immunosuppression executed by regulatory T_reg_ and increased levels or pro-inflammatory cytokines and chemokines. These effects are accomplished through action on the hypoxia/adenosine/A2AR immunosuppressive pathway, because they were not replicated in A2AR^−/−^ mice [22]. 

A2AR has been considered a target in anticancer immunotherapy. In a pharmacological intervention assay, the efficiency of reverting the immunosuppression of induced tumors with PD-1 antibodies (responsible for the immunological checkpoint [139]) improved, if an A2AR antagonist was co-administrated [140]. Another approach has consisted in evaluating the effect of A2AR deletion in cultured-activated tumor-draining lymph node (TDLN) T cells. In tissue lacking A2AR, tumor rejection improved, immunosuppression was diminished and the secretion of IFN-γ by T cells was enhanced [141]. Additionally, the ablation of ADO signaling promoted natural killer cell (NK) maturation and reduced tumor growth [142]. It has also been demonstrated in colorectal cancer cells that A2BR working synergically with A2AR, expressed in tumor-associated fibroblast, participated in the immune checkpoint dependent on NT5E/ADO to establish the immunosuppressive response characteristic of tumor cells [143].

Recently, it was reported that A1R deletion suppressed melanoma-derived cell growth and induced the inhibition of T cells in co-culture, antagonizing the anti-tumor immune response depending on another A2AR receptor, through a pathway involving overexpression of PD-L1 driven by the transcription factor ATF3 [144]. Taken together, these observations lead us to conclude that actions of ARs are too complex and could be opposite in diverse physiological events. Therefore, they need to be considered specifically. 

The immunosuppressive actions of ADO are orchestrated by tumor cells, but it has been proposed that this signaling can be amplified by influencing myeloid cell constituents of the TME, such as tumor-associated macrophages (TAMs). An interesting work has demonstrated that ADO generation by ovarian cancer cell lines attracts myeloid cells, inducing their differentiation in M2-TAM (macrophages with a non-inflammatory phenotype). Moreover, TAMs display an incremented expression of CD39 and stromal fibroblast (SF) for CD73; thus, TAMs and SF collaborate to amplify ADO formation and, consequently, the immunosuppressive effect [145].

In general, the actions of ADO inhibiting the anti-tumor immune response have been demonstrated in a broad group of host immune cells in the TME. An overview of these actions is presented in Table 1.

Since ADO accumulation in the TME has deleterious effects on immune surveillance, the ectonucleotidases (CD39/CD73) involved in their synthesis are an obvious target to unleash the immune inhibition executed by ADO. Recently developed antibodies targeting these enzymes were used to promote antitumor immunity, by targeting ectonucleotidase expression in DCs, macrophages and T cells [165]. Previous research has demonstrated the effect of antibodies against CD39 and CD73 in the immune response against ovarian cancer cell lines; it was found that NK and T cell cytotoxicity was improved and the proliferation of CD4^+^ T cells were uninhibited. These effects were achieved by a reduction in ADO synthesis [166]. Moreover, since focal radiotherapy induces overexpression of CD73 and, thus, an increment in ADO in the TME, blocking of CD73 has been assayed in combination with focal radiotherapy and immune checkpoint blockade (directed to cytotoxic T-lymphocyte-associated protein 4, PDL-1 and PD-1 in breast cancer cells); in these assays, CD73 blocks improved DC infiltration and the induction of anti-tumor T cell-dependent responses [167].

An important mechanism of interaction between tumor cells and TME and host is exosome-dependent signaling. Exosomes are signalosomes assembled in small vesicles (30-100 μm) that are released by exocytosis and induce cellular responses in the target. In cancer, exosomes have been characterized as entities carrying elements to induce EMT and metastasis, such as TGF-β and HIF-1α, as well as immunosuppressive elements [168]. 

The expression of CD39 and CD73 in cancer exosomes (CSE) was demonstrated in bladder cancer cells [169]. The ability to dephosphorylate ATP to form ADO was documented in exosomes from bladder (HT1376 line), colon cancer (CaCo line) and in malignant effusions of mesothelioma patients. ADO promoted a negative regulation of T cells in the TME [170]. It was shown that exosomes from the prostate carcinoma cell line DU145, expressing CD39 and CD73, inhibited DC activities that resulted in an immunosuppressive environment [171]. Thus, exosomes represent an important piece in the interaction and modification of the environment by purines in tumor cells.

Research is increasingly aimed at understanding the mechanism that regulates CD39 and CD73 expression. CD73 expression is regulated by a net of cellular messengers, transcription factors (TF) and miRNA [172]. Recently, it was shown that the P30 isoform—but not the wild-type version of CEPBA TF, a protein frequently mutated in acute myeloid leukemia (AML)—interacts with the promoter region of the NT5E gene in AML, to induce its expression and mediate AML progression via the NT5E-A2AR pathway [173]. In hepatic stellate cells, it has been shown that SMAD2, SMAD3, SMAD4 and SMAD5 and SP1 TF bind the CD73 gene promoter [174], demonstrating that TGF-β is a regulator of CD73 expression [175]. Additionally, it has been observed that HIF-1 binds the NT5E gene promoter, confirming that hypoxia is a strong regulator of immune checkpoints dependent on ADO [20]. In a model of induction and reversion of EMT in hepatocellular carcinoma, TNF-〈 induced overexpression of the NT5E gene and reversion of EMT downregulation [176]. In agreement, a bioinformatics analysis using the gene Signature Finder Algorithm (gSFA) found in colorectal cancer that NT5E belongs to the gene signature of this disease, and that it is a transcriptional target of TNF-〈 [177]. In Th17 cells, differentiated in vitro by a combination of IL-6 and TGF-®, IL-6 through Stat3 positively regulated NT5E expression, while TGF-β through Gfi-1 repressed its expression, but the cells displayed an immunosuppressive phenotype [178]. Moreover, the processing of RNAs coding for NT5E is regulated by a miRNA group, the presence of this miRNA contributes to the role of CD73 in cancer [170].

## 3. Concluding Remarks

Cancer is a complex disease; intense efforts have been made to understand and systematize the general principles underlying cancer cells identity and tumor-host interactions, to decipher how tumor cells self-regulate their differentiation, growth and expansion. For that, cancer hallmarks involve an important frame of reference, encompassing those characteristics that made cancer cells biologically successful [7,8]. In this review, we organized existing evidence showing that purinergic signaling is an important modulator in the acquisition and maintenance of cancer cell phenotypes, the establishment of their social interactions and bidirectional relationship with the environment. 

From the accumulate data, it is deduced that purinergic system in TME impacts tumor biology in two main ways: (1) exerting autocrine-paracrine actions over the own tumor cells, to establish a feedback loop that integrate energy metabolism with cellular tasks, such as cell proliferation, migration and metastatic induction; and (2) regulating the cellular interactions with the host, mainly by mediating a dialog with the immune system, to avoid a correct immunological response. Thus, purinergic signaling could be considered a master regulator of tumor cells identity and collective cellular properties.

A detailed knowledge of purinergic signaling elements and its mechanistic processes in the distinct level of cellular interactions in cancerous cells will be necessary to open new avenues in the search of therapeutic targets against carcinogenesis.

## Figures and Tables

**Table 1:** Summary of ADO’s actions on immune cells in the TME context.

Cell Type	Observations	References
T regulatory cells	CD39 and CD73 are markers of Foxp3^+^ T_reg_ which express A2AR. A2AR activity induces cell proliferation and PD-1 expression, promoting an anergic state. ADO stimulation creates a feedback loop that maintain a constant number of CD4^+^Foxp3^+^ T_reg_ in tumors to inhibit antitumor response. Blocking of A2AR increases CD8^+^ cells.	[146,147,148]
T effector cells	T_eff_ cells express CD73; its pharmacological inhibition with APCP induces increment of NFkB activity and IFNγ released by CD4^+^ T-cells. Stimulation of A2AR induces: 1) a marked reduction in IL-1, 2, 3, 4, 12 and 13, TNFα, IFNγ, GM-CSF, CCL3 and CCL4. 2) a reduction of CD8^+^ and CD4^+^ expansion by inhibition of cell proliferation. 3) a decrement of cytotoxic activity of CD8^+^ cells, and 4) T-cell apoptosis;	[149,150,151,152,153]
NK cells	ADO acting through A2AR, limits maturation of NK cells by suppressing cytotoxic activity and cytokine production. In NK cells positive to CD73, the expression of proteins related with immune check points as: LAG-3, VISTA, PD-1, and PD-L1 have higher expression; IL-10 is also up-regulated, producing inhibition of CD4^+^ T cells proliferation and IFNγ production. ADO acting through A2AR inhibits the cytotoxicity of activated NK cells.	[142,154,155,156]
Myeloid cells	In macrophages ADO acting by A2AR inhibits M-CSF dependent proliferation and suppresses IL-12 and TNF-α production. By A2BR induces IL-10 synthesis. CD14^+^ CD163^+^ -TAM, from ovarian cancer, express incremented levels of CD39 that modulates their immunosupresive functions; ectoenzyme expression is modulated by IL-27. In TME ADO attract myeloid cells, induces their differentiation in M2 macrophages to favor immune evasion. In hematopoietic cells, A2BR induce accumulation of immunosupressive MDSC. A2BR activity alters DC differentiation and induces generation of cells expressing suppressors of immune antitumor response.	[145,157,158,159,160,161,162,163,164]

## References

[1] Burnstock G. (2009). Purinergic signalling. Br. J. Pharmacol..

[2] Coddou C., Yan Z., Obsil T., Huidobro-Toro J.P., Stojilkovic S.S. (2011). Activation and regulation of purinergic P2X receptor channels. Pharmacol. Rev..

[3] Abbracchio M.P., Burnstock G., Boeynaems J.-M., Barnard E.A., Boyer J.L., Kennedy C., Knight G.E., Fumagalli M., Gachet C., Jacobson K.A. (2006). International Union of Pharmacology LVIII: Update on the P2Y G Protein-Coupled Nucleotide Receptors: From Molecular Mechanisms and Pathophysiology to Therapy. Pharmacol. Rev..

[4] Yegutkin G.G. (2014). Enzymes involved in metabolism of extracellular nucleotides and nucleosides: Functional implications and measurement of activities. Crit. Rev. Biochem. Mol. Biol..

[5] Stagg J., Smyth M.J. (2010). Extracellular adenosine triphosphate and adenosine in cancer. Oncogene.

[6] Corriden R., Insel P.A. (2010). Basal Release of ATP: An Autocrine-Paracrine Mechanism for Cell Regulation. Sci. Signal..

[7] Hanahan D., Weinberg R.A. (2000). The hallmarks of cancer. Cell.

[8] Hanahan D., Weinberg R.A. (2011). Hallmarks of cancer: The next generation. Cell.

[9] Balkwill F.R., Capasso M., Hagemann T. (2005). The tumor microenvironment at a glance. J. Cell Sci..

[10] Di Virgilio F., Adinolfi E. (2017). Extracellular purines, purinergic receptors and tumor growth. Oncogene.

[11] Di Virgilio F., Sarti A.C., Falzoni S., De Marchi E., Adinolfi E. (2018). Extracellular ATP and P2 purinergic signalling in the tumour microenvironment. Nat. Rev. Cancer.

[12] Volonté C., D’Ambrosi N. (2009). Membrane compartments and purinergic signalling: The purinome, a complex interplay among ligands, degrading enzymes, receptors and transporters. FEBS J..

[13] Gusovsky F., Daly J.W., Yasumoto T., Rojas E. (1988). Differential effects of maitotoxin on ATP secretion and on phosphoinositide breakdown in rat pheochromocytoma cells. FEBS Lett..

[14] Pedersen S., Pedersen S.F., Nilius B., Lambert I.H., Hoffmann E.K. (1999). Mechanical stress induces release of ATP from Ehrlich ascites tumor cells. Biochim. Biophys. Acta.

[15] Takai E., Tsukimoto M., Harada H., Sawada K., Moriyama Y., Kojima S. (2012). Autocrine regulation of TGF-β1-induced cell migration by exocytosis of ATP and activation of P2 receptors in human lung cancer cells. J. Cell Sci..

[16] Vázquez-Cuevas F.G., Martínez-Ramírez A.S., Robles-Martínez L., Garay E., García-Carrancá A., Pérez-Montiel D., Castañeda-García C., Arellano R.O. (2014). Paracrine stimulation of P2X7 receptor by ATP activates a proliferative pathway in ovarian carcinoma cells. J. Cell. Biochem..

[17] Liu H., Yuan M., Yao Y., Wu D., Dong S., Tong X. (2019). In Vitro effect of Pannexin 1 channel on the invasion and migration of I-10 testicular cancer cells via ERK1/2 signaling pathway. Biomed. Pharmacother..

[18] Pellegatti P., Raffaghello L., Bianchi G., Piccardi F., Pistoia V., Di Virgilio F. (2006). Increased level of extracellular ATP at tumor sites: In vivo imaging with plasma membrane luciferase. PLoS ONE.

[19] Ohta A., Gorelik E., Prasad S.J., Ronchese F., Lukashev D., Wong M.K., Huang X., Caldwell S., Liu K., Smith P. (2006). A2A adenosine receptor protects tumors from antitumor T cells. Proc. Natl. Acad. Sci. USA.

[20] Synnestvedt K., Furuta G.T., Comerford K.M., Louis N., Karhausen J., Eltzschig H.K., Hansen K.R., Thompson L.F., Colgan S.P. (2002). Ecto-5’-nucleotidase (CD73) regulation by hypoxia-inducible factor-1 mediates permeability changes in intestinal epithelia. J. Clin. Investig..

[21] Poth J.M., Brodsky K., Ehrentraut H., Grenz A., Eltzschig H.K. (2013). Transcriptional control of adenosine signaling by hypoxia-inducible transcription factors during ischemic or inflammatory disease. J. Mol. Med..

[22] Hatfield S.M., Kjaergaard J., Lukashev D., Schreiber T.H., Belikoff B., Abbott R., Sethumadhavan S., Philbrook P., Ko K., Cannici R. (2015). Immunological mechanisms of the antitumor effects of supplemental oxygenation. Sci. Transl. Med..

[23] Sitkovsky M.V., Hatfield S., Abbott R., Belikoff B., Lukashev D., Ohta A. (2014). Hostile, Hypoxia-A2-Adenosinergic Tumor Biology as the Next Barrier to Overcome for Tumor Immunologists. Cancer Immunol. Res..

[24] Allard B., Beavis P.A., Darcy P.K., Stagg J. (2016). Immunosuppressive activities of adenosine in cancer. Curr. Opin. Pharmacol..

[25] Furlow P.W., Zhang S., Soong T.D., Halberg N., Goodarzi H., Mangrum C., Wu Y.G., Elemento O., Tavazoie S.F. (2015). Mechanosensitive pannexin-1 channels mediate microvascular metastatic cell survival. Nat. Cell Biol..

[26] Gombault A., Baron L., Couillin I. (2012). ATP release and purinergic signaling in NLRP3 inflammasome activation. Front. Immunol..

[27] Mantel A., Harvey V. (2015). P2X7/PANX1 as a new target for melanoma?. Exp. Dermatol..

[28] Pelegrin P., Surprenant A. (2006). Pannexin-1 mediates large pore formation and interleukin-1beta release by the ATP-gated P2X7 receptor. EMBO J..

[29] De Leve S., Wirsdörfer F., Jendrossek V. (2019). Targeting the Immunomodulatory CD73/Adenosine System to Improve the Therapeutic Gain of Radiotherapy. Front Immunol..

[30] De Andrade Mello P., Coutinho-Silva R., Savio L. (2017). Multifaceted effects of extracellular adenosine triphosphate and adenosine in the tumor-host interaction and therapeutic perspectives. Front. Immunol..

[31] Coutinho-Silva R., Persechini P.M., Bisaggio R.D., Perfettini J.L., Neto A.C., Kanellopoulos J.M., Motta-Ly I., Dautry-Varsat A., Ojcius D.M. (1999). P2Z/P2X7 receptor-dependent apoptosis of dendritic cells. Am. J. Physiol..

[32] Costa-Junior H.M., Sarmento Vieira F., Coutinho-Silva R. (2011). C terminus of the P2X7 receptor: Treasure hunting. Purinergic Signal..

[33] Slater M., Danieletto S., Gidley-Baird A., Teh L.C., Barden J.A. (2004). Early prostate cancer detected using expression of non-functional cytolytic P2X7 receptors. Histopathology.

[34] Solini A., Cuccato S., Ferrari D., Santini E., Gulinelli S., Callegari M.G., Dardano A., Faviana P., Madec S., Di Virgilio F. (2008). Increased P2X7 receptor expression and function in thyroid papillary cancer: A new potential marker of the disease?. Endocrinology.

[35] Giannuzzo A., Pedersen S.F., Novak I. (2015). The P2X7 receptor regulates cell survival, migration and invasion of pancreatic ductal adenocarcinoma cells. Mol. Cancer.

[36] Qian F., Xiao J., Hu B., Sun N., Yin W., Zhu J. (2017). High expression of P2X7R is an independent postoperative indicator of poor prognosis in colorectal cancer. Hum. Pathol..

[37] Calik I., Calik M., Turken G., Ozercan I.H. (2020). A promising independent prognostic biomarker in colorectal cancer: P2X7 receptor. Int. J. Clin. Exp. Pathol..

[38] Asif A., Khalid M., Manzoor S., Ahmad H., Rehman A.U. (2019). Role of purinergic receptors in hepatobiliary carcinoma in Pakistani population: An approach towards proinflammatory role of P2X4 and P2X7 receptors. Purinergic Signal..

[39] Di Virgilio F., Ferrari D., Adinolfi E. (2009). P2X (7): A growth-promoting receptor-implications for cancer. Purinergic Signal..

[40] Adinolfi E., Callegari M.G., Ferrari D., Bolognesi C., Minelli M., Wieckowski M.R., Pinton P., Rizzuto R., Di Virgilio F. (2005). Basal activation of the P2X7 ATP receptor elevates mitochondrial calcium and potential, increases cellular ATP levels, and promotes serum-independent growth. Mol. Biol. Cell.

[41] Gilbert S.M., Oliphant C.J., Hassan S., Peille A.L., Bronsert P., Falzoni S., Di Virgilio F., McNulty S., Lara R. (2019). ATP in the tumour microenvironment drives expression of nfP2X7, a key mediator of cancer cell survival. Oncogene.

[42] Amoroso F., Salaro E., Falzoni S., Chiozzi P., Giuliani A.L., Cavallesco G., Maniscalco P., Puozzo A., Bononi I., Martini F. (2016). P2X7 targeting inhibits growth of human mesothelioma. Oncotarget.

[43] Choi J.H., Ji Y.G., Ko J.J., Cho H.J., Lee D.H. (2018). Activating P2X7 Receptors Increases Proliferation of Human Pancreatic Cancer Cells via ERK1/2 and JNK. Pancreas.

[44] Zhang Y., Cheng H., Li W., Wu H., Yang Y. (2019). Highly-expressed P2X7 receptor promotes growth and metastasis of human HOS/MNNG osteosarcoma cells via PI3K/Akt/GSK3β/β-catenin and mTOR/HIF1α/VEGF signaling. Int. J. Cancer.

[45] Bradford M.D., Soltoff S.P. (2002). P2X7 receptors activate protein kinase D and p42/p44 mitogen-activated protein kinase (MAPK) downstream of protein kinase C. Biochem. J..

[46] Stefano L., Rössler O.G., Griesemer D., Hoth M., Thiel G. (2007). P2X(7) receptor stimulation upregulates Egr-1 biosynthesis involving a cytosolic Ca(2+) rise, transactivation of the EGF receptor and phosphorylation of ERK and Elk-1. J. Cell Physiol..

[47] Greig A.V., Linge C., Healy V., Lim P., Clayton E., Rustin M.H., McGrouther D.A., Burnstock G. (2003). Expression of purinergic receptors in non-melanoma skin cancers and their functional roles in A431 cells. J. Invest. Dermatol..

[48] Aquea G., Bresky G., Lancellotti D., Madariaga J.A., Zaffiri V., Urzua U., Haberle S., Bernal G. (2014). Increased expression of P2RY2, CD248 and EphB1 in gastric cancers from Chilean patients. Asian Pac. J. Cancer Prev..

[49] Xie R., Xu J., Wen G., Jin H., Liu X., Yang Y., Ji B., Jiang Y., Song P., Dong H. (2014). The P2Y2 nucleotide receptor mediates the proliferation and migration of human hepatocellular carcinoma cells induced by ATP. J. Biol. Chem..

[50] Tu M.T., Luo S.F., Wang C.C., Chien C.S., Chiu C.T., Lin C.C., Yang C.M. (2000). P2Y(2) receptor-mediated proliferation of C(6) glioma cells via activation of Ras/Raf/MEK/MAPK pathway. Br. J. Pharmacol..

[51] Choi J.H., Ji Y.G., Lee D.H. (2013). Uridine triphosphate increases proliferation of human cancerous pancreatic duct epithelial cells by activating P2Y2 receptor. Pancreas.

[52] Hevia M.J., Castro P., Pinto K., Reyna-Jeldes M., Rodríguez-Tirado F., Robles-Planells C., Ramírez-Rivera S., Madariaga J.A., Gutierrez F., López J. (2019). Differential Effects of Purinergic Signaling in Gastric Cancer-Derived Cells Through P2Y and P2X Receptors. Front. Pharmacol..

[53] Draganov D., Gopalakrishna-Pillai S., Chen Y.R., Zuckerman N., Moeller S., Wang C., Ann D., Lee P.P. (2015). Modulation of P2X4/P2X7/Pannexin-1 sensitivity to extracellular ATP via Ivermectin induces a non-apoptotic and inflammatory form of cancer cell death. Sci. Rep..

[54] Li X., Qi X., Zhou L., Catera D., Rote N.S., Potashkin J., Abdul-Karim F.W., Gorodeski G.I. (2007). Decreased expression of P2X7 in endometrial epithelial pre-cancerous and cancer cells. Gynecol. Oncol..

[55] Fu W., McCormick T., Qi X., Luo L., Zhou L., Li X., Wang B.C., Gibbons H.E., Abdul-Karim F.W., Gorodeski G.I. (2009). Activation of P2X(7)-mediated apoptosis Inhibits DMBA/TPA-induced formation of skin papillomas and cancer in mice. BMC Cancer.

[56] Katzur A.C., Koshimizu T., Tomić M., Schultze-Mosgau A., Ortmann O., Stojilkovic S.S. (1999). Expression and responsiveness of P2Y2 receptors in human endometrial cancer cell lines. J. Clin. Endocrinol. Metab..

[57] Höpfner M., Maaser K., Barthel B., von Lampe B., Hanski C., Riecken E.O., Zeitz M., Scherübl H. (2001). Growth inhibition and apoptosis induced by P2Y2 receptors in human colorectal carcinoma cells: Involvement of intracellular calcium and cyclic adenosine monophosphate. Int. J. Colorectal Dis..

[58] Maaser K., Höpfner M., Kap H., Sutter A.P., Barthel B., von Lampe B., Zeitz M., Scherübl H. (2002). Extracellular nucleotides inhibit growth of human oesophageal cancer cells via P2Y(2)-receptors. Br. J. Cancer.

[59] Yang G., Zhang S., Zhang Y., Zhou Q., Peng S., Zhang T., Yang C., Zhu Z., Zhang F. (2014). The inhibitory effects of extracellular ATP on the growth of nasopharyngeal carcinoma cells via P2Y2 receptor and osteopontin. J. Exp. Clin. Cancer Res..

[60] Wan H., Xie R., Xu J., He J., Tang B., Liu Q., Wang S., Guo Y., Yang X., Dong T.X. (2017). Anti-proliferative Effects of Nucleotides on Gastric Cancer via a Novel P2Y6/SOCE/Ca^2+^/β-catenin Pathway. Sci. Rep..

[61] Xiang H.J., Liu Z.C., Wang D.S., Chen Y., Yang Y.L., Dou K.F. (2006). Adenosine A(2b) receptor is highly expressed in human hepatocellular carcinoma. Hepatol. Res..

[62] Ma D.F., Kondo T., Nakazawa T., Niu D.F., Mochizuki K., Kawasaki T., Yamane T., Katoh R. (2018). Hypoxia-inducible adenosine A2B receptor modulates proliferation of colon carcinoma cells. Hum. Pathol..

[63] Kasama H., Sakamoto Y., Kasamatsu A., Okamoto A., Koyama T., Minakawa Y., Ogawara K., Yokoe H., Shiiba M., Tanzawa H. (2015). Adenosine A2b receptor promotes progression of human oral cancer. BMC Cancer.

[64] Zhou Y., Chu X., Deng F., Tong L., Tong G., Yi Y., Liu J., Tang J., Tang Y., Xia Y. (2017). The adenosine A2b receptor promotes tumor progression of bladder urothelial carcinoma by enhancing MAPK signaling pathway. Oncotarget.

[65] Wei Q., Costanzi S., Balasubramanian R., Gao Z.G., Jacobson K.A. (2013). A2B adenosine receptor blockade inhibits growth of prostate cancer cells. Purinergic Signal..

[66] Fernandez-Gallardo M., González-Ramírez R., Sandoval A., Felix R., Monjaraz E. (2016). Adenosine Stimulate Proliferation and Migration in Triple Negative Breast Cancer Cells. PLoS ONE.

[67] Wilkat M., Bast H., Drees R., Dünser J., Mahr A., Azoitei N., Marienfeld R., Frank F., Brhel M., Ushmorov A. (2020). Adenosine receptor 2B activity promotes autonomous growth, migration as well as vascularization of head and neck squamous cell carcinoma cells. Int. J. Cancer.

[68] Fife C.M., McCarroll J.A., Kavallaris M. (2014). Movers and shakers: Cell cytoskeleton in cancer metastasis. Br. J. Pharmacol..

[69] Martínez-Ramírez A.S., Díaz-Muñoz M., Butanda-Ochoa A., Vázquez-Cuevas F.G. (2017). Nucleotides and nucleoside signaling in the regulation of the epithelium to mesenchymal transition (EMT). Purinergic Signal..

[70] Lu W., Kang Y. (2019). Epithelial-Mesenchymal plasticity in cancer progression and metastasis. Dev. Cell.

[71] Cao Y., Wang X., Li Y., Evers M., Zhang H., Chen X. (2019). Extracellular and macropinocytosis internalized ATP work together to induce epithelial–mesenchymal transition and other early metastatic activities in lung cancer. Cancer Cell Int..

[72] Takai E., Tsukimoto M., Harada H., Kojima S. (2014). Autocrine signaling via release of ATP and activation of P2X7 receptor influences motile activity of human lung cancer cells. Purinergic Signal..

[73] Qiu Y., Li W.H., Zhang H.Q., Liu Y., Tian X.X., Fang W.G. (2014). P2X7 mediates ATP-driven invasiveness in prostate cancer cells. PLoS ONE.

[74] Xia J., Yu X., Tang L., Li G., He T. (2015). P2X7 receptor stimulates breast cancer cell invasion and migration via the AKT pathway. Oncol. Rep..

[75] Ji Z., Xie Y., Guan Y., Zhang Y., Cho K.S., Ji M., You Y. (2018). Involvement of P2X7 Receptor in proliferation and migration of human glioma Cells. Biomed Res. Int..

[76] Ziberi S., Zuccarini M., Carluccio M., Giuliani P., Ricci-Vitiani L., Pallini R., Caciagli F., Di Iorio P., Ciccarelli R. (2019). Upregulation of epithelial-to-mesenchymal transition markers and P2X7 receptors is associated to increased invasiveness caused by P2X7 receptor stimulation in human glioblastoma stem cells. Cells.

[77] Bagchi S., Liao Z., Gonzalez F.A., Chorna N.E., Seye C.I., Weisman G.A., Erb L. (2005). The P2Y2 nucleotide receptor interacts with alphav integrins to activate Go and induce cell migration. J. Biol. Chem..

[78] Liao Z., Seye C.I., Weisman G.A., Erb L. (2007). The P2Y2 nucleotide receptor requires interaction with alpha v integrins to access and activate G12. J. Cell Sci..

[79] Jin H., Eun S.Y., Lee J.S., Park S.W., Lee J.H., Chang K.C., Kim H.J. (2014). P2Y2 receptor activation by nucleotides released from highly metastatic breast cancer cells increases tumor growth and invasion via crosstalk with endothelial cells. Breast Cancer Res..

[80] Chen L., He H.Y., Li H.M., Zheng J., Heng W.J., You J.F., Fang W.G. (2004). ERK1/2 and p38 pathways are required for P2Y receptor-mediated prostate cancer invasion. Cancer Lett..

[81] Zhang Y., Gong L.H., Zhang H.Q., Du Q., You J.F., Tian X.X., Fang W.G. (2010). Extracellular ATP enhances in vitro invasion of prostate cancer cells by activating Rho GTPase and upregulating MMPs expression. Cancer Lett..

[82] Li W.-H., Qiu Y., Zhang H.-Q., Liu Y., You J.-F., Tian X.-X., Fang W.-G. (2013). P2Y2 receptor promotes cell invasion and metastasis in prostate cancer cells. Br. J. Cancer.

[83] Chadet S., Jelassi B., Wannous R., Angoulvant D., Chevalier S., Besson P., Roger S. (2014). The activation of P2Y2 receptors increases MCF-7 breast cancer cells migration through the MEK-ERK1/2 signalling pathway. Carcinogenesis.

[84] Qiu Y., Liu Y., Li W.-H., Zhang H.-Q., Tian X.-X., Fang W.-G. (2018). P2Y2 receptor promotes the migration and invasion of breast cancer cells via EMT-related genes Snail and E-cadherin. Oncol. Rep..

[85] Zhang J., Liu Y., Yang H., Zhang H., Tian X., Fang W. (2017). ATP-P2Y2-β-catenin axis promotes cell invasion in breast cancer cells. Cancer Sci..

[86] Eun S.Y., Ko Y.S., Park S.W., Chang K.C., Kim H.J. (2015). P2Y2 nucleotide receptor-mediated extracellular signal-regulated kinases and protein kinase C activation induces the invasion of highly metastatic breast cancer cells. Oncol. Rep..

[87] Li W.H., Qiu Y., Zhang H.Q., Tian X.X., Fang W.G. (2015). P2Y2 Receptor and EGFR Cooperate to Promote Prostate Cancer Cell Invasion via ERK1/2 Pathway. PLoS ONE.

[88] Martínez-Ramírez A.S., Garay E., García-Carrancá A., Vázquez-Cuevas F.G. (2016). The P2RY2 Receptor Induces carcinoma cell migration and EMT through Cross- Talk With epidermal growth factor receptor. J. Cell. Biochem..

[89] Zhou P., Zhi X., Zhou T., Chen S., Li X., Wang L., Yin L., Shao Z., Ou Z. (2007). Overexpression of Ecto-5’-nucleotidase (CD73) promotes T-47D human breast cancer cells invasion and adhesion to extracellular matrix. Cancer Biol. Ther..

[90] Wang L., Zhou X., Zhou T., Ma D., Chen S., Zhi X., Yin L., Shao Z., Ou Z., Zhou P. (2008). Ecto-5’-nucleotidase promotes invasion, migration and adhesion of human breast cancer cells. J. Cancer Res. Clin. Oncol..

[91] Gao Z.W., Wang H.P., Dong K., Lin F., Wang X., Zhang H.Z. (2016). Adenosine inhibits migration, invasion and induces apoptosis of human cervical cancer cells. Neoplasma.

[92] Bowser J.L., Broaddus R.R. (2016). CD73s protection of epithelial integrity: Thinking beyond the barrier. Tissue Barriers.

[93] Ren Z.H., Lin C.Z., Cao W., Yang R., Lu W., Liu Z.Q., Chen Y.M., Yang X., Tian Z., Wang L.Z. (2016). CD73 is associated with poor prognosis in HNSCC. Oncotarget.

[94] Lupia M., Angiolini F., Bertalot G., Freddi S., Sachsenmeier K.F., Chisci E., Kutryb-Zajac B., Confalonieri S., Smolenski R.T., Giovannoni R. (2018). CD73 Regulates Stemness and epithelial-mesenchymal transition in ovarian cancer-initiating cells. Stem Cell Rep..

[95] Ma X.-L., Shen M.-N., Hu B., Wang B.-L., Yang W.-J., Lv L.-H., Wang H., Zhou Y., Jin A.L., Sun Y.F. (2019). CD73 promotes hepatocellular carcinoma progression and metastasis via activating PI3K/AKT signaling by inducing Rap1-mediated membrane localization of P110β and predicts poor prognosis. J. Hematol. Oncol..

[96] Virtanen S.S., Kukkonen-Macchi A., Vainio M., Elima K., Härkönen P.L., Jalkanen S., Yegutkin G.G. (2014). Adenosine inhibits tumor cell invasion via receptor-independent mechanisms. Mol. Cancer Res..

[97] Martínez-Ramírez A.S., Díaz-Muñoz M., Battastini A.M., Campos-Contreras A., Olvera A., Bergamin L., Glaser T., Jacintho Moritz C.E., Ulrich H., Vázquez-Cuevas F.G. (2017). Cellular Migration Ability Is Modulated by Extracellular Purines in Ovarian Carcinoma SKOV-3 Cells. J. Cell. Biochem..

[98] Zhou Y., Tong L., Chu X., Deng F., Tang J., Tang Y., Dai Y. (2017). The Adenosine A1 receptor antagonist DPCPX Inhibits tumor progression via the ERK/JNK Pathway in renal cell carcinoma. Cell Physiol. Biochem..

[99] Shi L., Wu Z., Miao J., Du S., Ai S., Xu E., Feng M., Song J., Guan W. (2019). Adenosine interaction with adenosine receptor A2a promotes gastric cancer metastasis by enhancing PI3K-AKT-mTOR signaling. Mol. Biol. Cell.

[100] Giacomelli C., Daniele S., Romei C., Tavanti L., Neri T., Piano I., Celi A., Martini C., Trincavelli M.L. (2018). The A_2B_ Adenosine receptor modulates the epithelial- mesenchymal transition through the balance of cAMP/PKA and MAPK/ERK Pathway activation in human epithelial lung cells. Front. Pharmacol..

[101] Yi Y., Zhou Y., Chu X., Zheng X., Fei D., Lei J., Qi H., Dai Y. (2020). Blockade of Adenosine A2b Receptor Reduces Tumor Growth and Migration in Renal Cell Carcinoma. J. Cancer.

[102] Jajoo S., Mukherjea D., Watabe K., Ramkumar V. (2009). Adenosine A(3) receptor suppresses prostate cancer metastasis by inhibiting NADPH oxidase activity. Neoplasia (N. Y.).

[103] Ledderose C., Hefti M.M., Chen Y., Bao Y., Seier T., Li L., Woehrle T., Zhang J., Junger W.G. (2016). Adenosine arrests breast cancer cell motility by A3 receptor stimulation. Purinergic Signal..

[104] Marucci G., Santinelli C., Buccioni M., Navia A.M., Lambertucci C., Zhurina A., Yli-Harja O., Volpini R., Kandhavelu M. (2018). Anticancer activity study of A_3_ adenosine receptor agonists. Life Sci..

[105] Torres Á., Erices J.I., Sanchez F., Ehrenfeld P., Turchi L., Virolle T., Uribe D., Niechi I., Spichiger C., Rocha J.D. (2019). Extracellular adenosine promotes cell migration/invasion of Glioblastoma Stem-like Cells through A_3_ Adenosine Receptor activation under hypoxia. Cancer Lett..

[106] Zanotelli M.R., Goldblatt Z.E., Miller J.P., Bordeleau F., Li J., VanderBurgh J.A., Lampi M.C., King M.R., Reinhart-King C.A. (2018). Regulation of ATP utilization during metastatic cell migration by collagen architecture. Mol. Biol. Cell.

[107] Kelley L.C., Chi Q., Cáceres R., Hastie E., Schindler A.J., Jiang Y., Matus D.Q., Plastino J., Sherwood D.R. (2019). Adaptive F-Actin polymerization and localized ATP production drive basement membrane invasion in the absence of MMPs. Dev. Cell.

[108] Warburg O. (1925). The metabolism of carcinoma cells. J. Cancer Res..

[109] Chen X., Qian Y., Wu S. (2015). The Warburg effect: Evolving interpretation of an established concept. Free Radic. Biol. Med..

[110] Liberti M.V., Locasale J.W. (2016). The Warburg Effect: How Does it Benefit Cancer Cells?. Trends Biochem. Sci..

[111] Israël M., Schwartz L. (2011). The metabolic advantage of tumor cells. Mol. Cancer.

[112] Schwartz L., Supuran C.T., Alfarouk K.O. (2017). The Warburg effect and the hallmarks of cancer. Anticancer Agents Med. Chem..

[113] Sawayama H., Ishimoto T., Sugihara H., Miyanari N., Miyamoto Y., Baba Y., Yoshida N., Baba H. (2014). Clinical impact of the Warburg effect in gastrointestinal cancer. Int. J. Oncol..

[114] Méndez I., Díaz-Muñoz M. (2018). Circadian and metabolic perspectives in the role played by NADPH in cancer. Front. Endocrinol..

[115] Scalise M., Pochini L., Galluccio M., Console L., Indiveri C. (2017). Glutamine transport and mitochondrial metabolism in cancer cell growth. Front. Oncol..

[116] Corbet C., Feron O. (2011). Cancer cell metabolism and mitochondria: Nutrient plasticity for TCA cycle fueling. Biochim. Biophys. Acta Rev. Cancer.

[117] De la Cruz López K.G., Toledo Guzmán M.E., Sánchez E.O., García Carrancá A. (2019). mTORC1 as a regulator of mitochondrial functions and a therapeutic target in cancer. Front. Oncol..

[118] Supplie L.M., Düking T., Campbell G., Diaz F., Moraes C.T., Götz M., Hamprecht B., Boretius S., Mahad D., Nave K.A. (2007). Respiration-deficient astrocytes survive as glycolytic cells in vivo. J. Neurosci..

[119] Potter M., Badder L., Hoade Y., Johnston I.G., Morten K.J. (2016). Monitoring intracellular oxygen concentration: Implications for hypoxia studies and real-time oxygen monitoring. Adv. Exp. Med. Biol..

[120] Baricordi O.R., Ferrari D., Melchiorri L., Chiozzi P., Hanau S., Chiari E., Rubini M., Di Virgilio F. (1996). An ATP-activated channel is involved in mitogenic stimulation of human T lymphocytes. Blood.

[121] Humphreys B.D., Rice J., Kertesy S.B., Dubyak G.R. (2000). Stress-activated protein kinase/JNK activation and apoptotic induction by the macrophage P2X7 nucleotide receptor. J. Biol. Chem..

[122] Graves L.M., Guy H.I., Kozlowski P., Huang M., Lazarowski E., Pope R.M., Collins M.A., Dahlstrand E.N., Earp H.S., Evans D.R. (2000). Regulation of carbamoyl phosphate synthetase by MAP kinase. Nature.

[123] Adinolfi E., Melchiorri L., Falzoni S., Chiozzi P., Morelli A., Tieghi A., Cuneo A., Castoldi G., Di Virgilio F., Baricordi O.R. (2002). P2X7 receptor expression in evolutive and indolent forms of chronic B lymphocytic leukemia. Blood.

[124] Amoroso F., Falzoni S., Adinolfi E., Ferrari E., Di Virgilio F. (2012). The P2X7 receptor is a key regulator of aerobic glycolysis. Cell Death Dis..

[125] Adinolfi E., Pizzirani C., Idzko M., Panther E., Norgauer J., Di Virgilio F., Ferrari D. (2005). P2X(7) receptor: Death or life?. Purinergic signal..

[126] Qian Y., Wang X., Liu Y., Li Y., Colvin R.A., Tong L., Wu S., Chen X. (2014). Extracellular ATP is internalized by macropinocytosis and induces intracellular ATP increase and drug resistance in cancer cells. Cancer Lett..

[127] Magnifico M.C., Macone A., Marani M., Bouzidi A., Giardina G., Rinaldo S., Cutruzzolà F., Paone A. (2019). Linking inflammation and prostate cancer progression: Toll-like receptor 3 stimulation rewires glucose metabolism in prostate cells. Anticancer Res..

[128] Murín R., Vidomanová E., Kowtharapu B.S., Hatok J., Dobrota D. (2017). Role of S-adenosylmethionine cycle in carcinogenesis. Gen. Physiol. Biophys..

[129] Mediavilla-Varela M., Luddy K., Noyes D., Khalil F.K., Neuger A.M., Soliman H., Antonia S.J. (2018). Antagonism of adenosine A2A receptor expressed by lung adenocarcinoma tumor cells and cancer associated fibroblasts inhibits their growth. Cancer Biol. Ther..

[130] Ledderose C., Woehrle T., Ledderose S., Strasser K., Seist R., Bao Y., Zhang J., Junger W.G. (2016). Cutting of the power: Inhibition of leukemia cell growth by pausing ATP release and P2X receptor signaling?. Purinergic Signal..

[131] Whiteside T.L. (2017). Targeting adenosine in cancer immunotherapy: A review of recent progress. Expert Rev. Anticancer Ther..

[132] Chekeni F.B., Elliott M.R., Sandilos J.K., Walk S.F., Kinchen J.M., Lazarowski E.R., Armstrong A.J., Penuela S., Laird D.W., Salvesen G.S. (2010). Pannexin 1 channels mediate «find-me» signal release and membrane permeability during apoptosis. Nature.

[133] Elliott M.R., Chekeni F.B., Trampont P.C., Lazarowski E.R., Kadl A., Walk S.F., Park D., Woodson R.I., Ostankovich M., Sharma P. (2009). Nucleotides released by apoptotic cells act as a find-me signal to promote phagocytic clearance. Nature.

[134] Trautmann A. (2009). Extracellular ATP in the immune system: More than just a «danger signal». Sci. Signal..

[135] Ghiringhelli F., Apetoh L., Tesniere A., Aymeric L., Ma Y., Ortiz C., Vermaelen K., Panaretakis T., Mignot G., Ullrich E. (2009). Activation of the NLRP3 inflammasome in dendritic cells induces IL-1beta-dependent adaptive immunity against tumors. Nat. Med..

[136] Aymeric L., Apetoh L., Ghiringhelli F., Tesniere A., Martins I., Kroemer G., Smyth M.J., Zitvogel L. (2010). Tumor cell death and ATP release prime dendritic cells and efficient anticancer immunity. Cancer Res..

[137] De Marchi E., Orioli E., Pegoraro A., Sangaletti S., Portararo P., Curti A., Colombo M.P., Di Virgilio F., Adinolfi E. (2019). The P2X7 receptor modulates immune cells infiltration, ectonucleotidases expression and extracellular ATP levels in the tumor microenvironment. Oncogene.

[138] Lecciso M., Ocadlikova D., Sangaletti S., Trabanelli S., De Marchi E., Orioli E., Pegoraro A., Portararo P., Jandus C., Bontadini A. (2017). ATP Release from Chemotherapy-Treated Dying Leukemia Cells Elicits an Immune Suppressive Effect by Increasing Regulatory T Cells and Tolerogenic Dendritic Cells. Front. Immunol..

[139] Chen L., Han X. (2015). Anti-PD-1/PD-L1 therapy of human cancer: Past, present, and future. J Clin. Invest..

[140] Beavis P.A., Milenkovski N., Henderson M.A., John L.B., Allard B., Loi S., Kershaw M.H., Stagg J., Darcy P.K. (1996). Adenosine receptor 2A blockade increases the efficacy of anti-PD-1 through enhanced antitumor T-cell responses. Cancer Immunol. Res..

[141] Kjaergaard J., Hatfield S., Jones G., Ohta A., Sitkovsky M. (2018). A2A Adenosine receptor gene deletion or Synthetic A2A Antagonist liberate Tumor-ReactiveCD8+ T Cells from tumor-induced immunosuppression. J. Immunol..

[142] Young A., Ngiow S.F., Gao Y., Patch A.-M., Barkauskas D.S., Messaoudene M., Lin G., Coudert J.D., Stannard K.A., Zitvogel L. (2018). A2AR Adenosine Signaling Suppresses Natural Killer Cell Maturation in the Tumor Microenvironment. Cancer Res..

[143] Yu M., Guo G., Huang L., Deng L., Chang C.S., Achyut B.R., Canning M., Xu N., Arbab A.S., Bollag R.J. (2020). CD73 on cancer-associated fibroblasts enhanced by the A_2B_-mediated feedforward circuit enforces an immune checkpoint. Nat. Commun..

[144] Liu H., Kuang X., Zhang Y., Ye Y., Li J., Liang L., Xie Z., Weng L., Guo J., Li H. (2020). ADORA1 Inhibition Promotes Tumor Immune Evasion by Regulating the ATF3-PD-L1 Axis. Cancer Cell.

[145] Montalbán Del Barrio I., Penski C., Schlahsa L., Stein R.G., Diessner J., Wöckel A., Dietl J., Lutz M.B., Mittelbronn M., Wischhusen J. (2016). Adenosine-generating ovarian cancer cells attract myeloid cells which differentiate into adenosine-generating tumor associated macrophages-a self-amplifying, CD39- and CD73-dependent mechanism for tumor immune escape. J. Immunother. Cancer.

[146] Deaglio S., Dwyer K.M., Gao W., Friedman D., Usheva A., Erat A., Chen J.F., Enjyoji K., Linden J., Oukka M. (2007). Adenosine generation catalyzed by CD39 and CD73 expressed on regulatory T cells mediates immune suppression. J. Exp. Med..

[147] Kinsey G.R., Huang L., Jaworska K., Khutsishvili K., Becker D.A., Ye H., Lobo P.I., Okusa M.D. (2012). Autocrine adenosine signaling promotes regulatory T cell-mediated renal protection. J. Am. Soc. Nephrol..

[148] Ma S.R., Deng W.W., Liu J.F., Mao L., Yu G.T., Bu L.L., Kulkarni A.B., Zhang W.F., Sun Z.J. (2017). Blockade of adenosine A2A receptor enhances CD8^+^ T cells response and decreases regulatory T cells in head and neck squamous cell carcinoma. Mol. Cancer.

[149] Zarek P.E., Huang C.T., Lutz E.R., Kowalski J., Horton M.R., Linden J., Drake C.G., Powell J.D. (2008). A2A receptor signaling promotes peripheral tolerance by inducing T-cell anergy and the generation of adaptive regulatory T cells. Blood.

[150] Erdmann A.A., Gao Z.G., Jung U., Foley J., Borenstein T., Jacobson K.A., Fowler D.H. (2005). Activation of Th1 and Tc1 cell adenosine A2A receptors directly inhibits IL-2 secretion in vitro and IL-2-driven expansion in vivo. Blood.

[151] Csóka B., Himer L., Selmeczy Z., Vizi E.S., Pacher P., Ledent C., Deitch E.A., Spolarics Z., Németh Z.H., Haskó G. (2008). Adenosine A2A receptor activation inhibits T helper 1 and T helper 2 cell development and effector function. FASEB J. Off. Publ. Fed. Am. Soc. Exp. Biol..

[152] Jin D., Fan J., Wang L., Thompson L.F., Liu A., Daniel B.J., Shin T., Curiel T.J., Zhang B. (2010). CD73 on tumor cells impairs antitumor T-cell responses: A novel mechanism of tumor-induced immune suppression. Cancer Res..

[153] Romio M., Reinbeck B., Bongardt S., Hüls S., Burghoff S., Schrader J. (2011). Extracellular purine metabolism and signaling of CD73-derived adenosine in murine Treg and Teff cells. Am. J. Physiol. Cell Physiol..

[154] Raskovalova T., Huang X., Sitkovsky M., Zacharia L.C., Jackson E.K., Gorelik E. (2005). Gs protein-coupled adenosine receptor signaling and lytic function of activated NK cells. J. Immunol..

[155] Raskovalova T., Lokshin A., Huang X., Jackson E.K., Gorelik E. (2005). Adenosine-mediated inhibition of cytotoxic activity and cytokine production by IL-2/NKp46-activated NK cells: Involvement of protein kinase A isozyme I (PKA I). Immunol. Res..

[156] Neo S.Y., Yang Y., Record J., Ma R., Chen X., Chen Z., Tobin N.P., Blake E., Seitz C., Thomas R. (2020). CD73 immune checkpoint defines regulatory NK cells within the tumor microenvironment. J. Clin. Invest..

[157] Xaus J., Valledor A.F., Cardó M., Marquès L., Beleta J., Palacios J.M., Celada A. (1999). Adenosine inhibits macrophage colony-stimulating factor-dependent proliferation of macrophages through the induction of p27kip-1 expression. J. Immunol..

[158] Haskó G., Kuhel D.G., Chen J.F., Schwarzschild M.A., Deitch E.A., Mabley J.G., Marton A., Szabó C. (2000). Adenosine inhibits IL-12 and TNF-[alpha] production via adenosine A2a receptor-dependent and independent mechanisms. FASEB J..

[159] Németh Z.H., Lutz C.S., Csóka B., Deitch E.A., Leibovich S.J., Gause W.C., Tone M., Pacher P., Vizi E.S., Haskó G. (2005). Adenosine augments IL-10 production by macrophages through an A2B receptor-mediated posttranscriptional mechanism. J. Immonol..

[160] Kreckler L.M., Wan T.C., Ge Z.D., Auchampach J.A. (2006). Adenosine inhibits tumor necrosis factor-alpha release from mouse peritoneal macrophages via A2A and A2B but not the A3 adenosine receptor. J. Pharmacol. Exp. Ther..

[161] Novitskiy S.V., Ryzhov S., Zaynagetdinov R., Goldstein A.E., Huang Y., Tikhomirov O.Y., Blackburn M.R., Biaggioni I., Carbone D.P., Feoktistov I. (2008). Adenosine receptors in regulation of dendritic cell differentiation and function. Blood.

[162] Ryzhov S., Novitskiy S.V., Goldstein A.E., Biktasova A., Blackburn M.R., Biaggioni I., Dikov M.M., Feoktistov I. (2011). Adenosinergic regulation of the expansion and immunosuppressive activity of CD11b+Gr1+ cells. J. Immunol..

[163] D’Almeida S.M., Kauffenstein G., Roy C., Basset L., Papargyris L., Henrion D., Catros V., Ifrah N., Descamps P., Croue A. (2016). The ecto-ATPDase CD39 is involved in the acquisition of the immunoregulatory phenotype by M-CSF-macrophages and ovarian cancer tumor-associated macrophages: Regulatory role of IL-27. Oncoimmunology.

[164] Li J., Wang L., Chen X., Li L., Li Y., Ping Y., Huang L., Yue D., Zhang Z., Wang F. (2017). CD39/CD73 upregulation on myeloid-derived suppressor cells via TGF-β-mTOR-HIF-1 signaling in patients with non-small cell lung cancer. Oncoimmunology.

[165] Perrot I., Michaud H.-A., Giraudon-Paoli M., Augier S., Docquier A., Gros L., Courtois R., Déjou C., Jecko D., Becquart O. (2019). Blocking Antibodies Targeting the CD39/CD73 Immunosuppressive Pathway Unleash Immune Responses in Combination Cancer Therapies. Cell Rep..

[166] Häusler S.F., Del Barrio I.M., Diessner J., Stein R.G., Strohschein J., Hönig A., Dietl J., Wischhusen J. (2014). Anti-CD39 and anti-CD73 antibodies A1 and 7G2 improve targeted therapy in ovarian cancer by blocking adenosine-dependent immune evasion. Am. J. Transl. Res..

[167] Wennerberg E., Spada S., Rudqvist N.P., Lhuillier C., Gruber S., Chen Q., Zhang F., Zhou X.K., Gross S.S., Formenti S.C. (2020). CD73 Blockade Promotes Dendritic Cell Infiltration of Irradiated Tumors and Tumor Rejection. Cancer Immunol. Res..

[168] Syn N., Wang L., Sethi G., Thiery J.P., Goh B.C. (2016). Exosome-Mediated Metastasis: From Epithelial-Mesenchymal Transition to Escape from Immunosurveillance. Trends Pharmacol. Sci..

[169] Welton J.L., Khanna S., Giles P.J., Brennan P., Brewis I.A., Staffurth J., Mason M.D., Clayton A. (2010). Proteomics analysis of bladder cancer exosomes. Mol. Cell Proteom..

[170] Clayton S.M., Archard J.A., Wagner J., Farwell D.G., Bewley A.F., Beliveau A., Birkeland A., Rao S., Abouyared M., Belafsky P.C. (2020). Immunoregulatory Potential of Exosomes Derived from Cancer Stem Cells. Stem Cells Dev..

[171] Salimu J., Webber J., Gurney M., Al-Taei S., Clayton A., Tabi Z. (2017). Dominant immunosuppression of dendritic cell function by prostate-cancer-derived exosomes. J. Extracell. Vesicles.

[172] Kordaß T., Osen W., Eichmüller S.B. (2018). Controlling the Immune Suppressor: Transcription Factors and MicroRNAs Regulating CD73/NT5E. Front. Immunol..

[173] Jakobsen J.S., Laursen L.G., Schuster M.B., Pundhir S., Schoof E., Ge Y., d’Altri T., Vitting-Seerup K., Rapin N., Gentil C. (2019). Mutant CEBPA directly drives the expression of the targetable tumor-promoting factor CD73 in AML. Sci. Adv..

[174] Fausther M., Sheung N., Saiman Y., Bansal M.B., Dranoff J.A. (2012). Activated hepatic stellate cells upregulate transcription of ecto-5’-nucleotidase/CD73 via specific SP1 and SMAD promoter elements. Am. J. Physiol. Gastrointest. Liver Physiol..

[175] Cappelli C., Tellez A., Jara C., Alarcón S., Torres A., Mendoza P., Podestá L., Flores C., Quezada C., Oyarzún C. (2020). The TGF-β profibrotic cascade targets ecto-5’-nucleotidase gene in proximal tubule epithelial cells and is a traceable marker of progressive diabetic kidney disease. Biochim. Biophys. Acta Mol. Basis. Dis..

[176] Shrestha R., Bridle K.R., Crawford D., Jayachandran A. (2019). TNF-α-mediated epithelial-to-mesenchymal transition regulates expression of immune checkpoint molecules in hepatocellular carcinoma. Mol. Med. Rep..

[177] Pagnotta S.M., Laudanna C., Pancione M., Sabatino L., Votino C., Remo A., Cerulo L., Zoppoli P., Manfrin E., Colantuoni V. (2013). Ensemble of gene signatures identifies novel biomarkers in colorectal cancer activated through PPARγ and TNFα signaling. PLoS ONE.

[178] Chalmin F., Mignot G., Bruchard M., Chevriaux A., Végran F., Hichami A., Ladoire S., Derangère V., Vincent J., Masson D. (2012). Stat3 and Gfi-1 transcription factors control Th17 cell immunosuppressive activity via the regulation of ectonucleotidase expression. Immunity.

